# Comparative Effectiveness of Continuous Subcutaneous Insulin Infusion Versus Multiple Daily Injections on Glycemic Control in Pediatric Type 1 Diabetes: A Systematic Review, Meta-Analysis, and Trial Sequential Analysis

**DOI:** 10.14740/jocmr6560

**Published:** 2026-08-26

**Authors:** Zeinh Hussein Fardan, Alam Eldin Musa Mustafa, Niemat Mohammed Tahir Ali, Youssef A. Alqahtani, Marwa Hussein Said Ahmad Alhag, Leen A. Aburas, Mai Mazen Alzayer, Khulud Musallam Salem Alsulami, Lama Atef, Ali Said Ali Metwaly

**Affiliations:** a Department of Pediatrics and Child Health, College of Medicine, King Khalid University, Abha, Saudi Arabia; b Maternity and Child Hospital, Hail, Saudi Arabia; c College of Medicine, Istinye Universitesi, Istanbul, Turkey; d Ibn Sina National College, Dammam, Saudi Arabia; e University of Jeddah, Jeddah, Saudi Arabia; f College of Medicine, Alfaisal University, Riyadh, Saudi Arabia; g Precision Medicine, Faculty of Pharmacy, Alexandria University, Alexandria, Egypt

**Keywords:** Type 1 diabetes mellitus, Pediatrics, Continuous subcutaneous insulin infusion, Multiple daily injections, Meta-analysis, Trial sequential analysis, Glycemic control

## Abstract

**Background:**

Intensive insulin therapy is crucial for mitigating complications in pediatric type 1 diabetes mellitus (T1DM). This study aimed to evaluate the impact of continuous subcutaneous insulin infusion (CSII) versus multiple daily injections (MDI) on glycemic control and safety in pediatric patients.

**Methods:**

A systematic search of PubMed/MEDLINE, Embase, and Cochrane Central was conducted for randomized controlled trials (RCTs) published up to 2026. Data were synthesized using random-effects meta-analysis to calculate mean differences (MD) for hemoglobin A1c (HbA1c) and risk ratios (RR) for adverse events. Trial sequential analysis (TSA) was employed to assess the sufficiency of evidence. Certainty of evidence was graded using the Grades of Recommendations Assessment, Development, and Evaluation (GRADE) approach.

**Results:**

Fourteen RCTs involving 659 participants were included. CSII was associated with a modest reduction in HbA1c compared to MDI (MD −0.36%; 95% confidence interval (CI), −0.80 to 0.08), although this did not reach statistical significance in the primary analysis (P = 0.098). However, sensitivity analysis excluding one large pragmatic trial revealed a significant benefit for CSII (MD −0.27%, P < 0.0001). No significant differences were found in the risks of severe hypoglycemia (RR = 0.83) or diabetic ketoacidosis (RR = 1.40). TSA indicated that the required information size for a definitive conclusion on HbA1c has not yet been met.

**Conclusions:**

CSII may offer a modest glycemic advantage over MDI in pediatric T1DM without increasing safety risks; however, current randomized evidence is insufficient to universally recommend one modality over the other. Treatment choice should be personalized. Although the proportion of patients using automated insulin delivery (AID) is rising rapidly, CSII retains practical and cost advantages; future research should focus on modern AID systems.

## Introduction

Type 1 diabetes mellitus (T1DM) is one of the most prevalent chronic autoimmune diseases in childhood and adolescence, characterized by a rising incidence rate [1, 2]. As established by the landmark Diabetes Control and Complications Trial (DCCT), intensive glycemic control is paramount to delaying the onset and mitigating the progression of long-term microvascular and macrovascular complications [3, 4]. However, achieving and maintaining optimal hemoglobin A1c (HbA1c) targets is a clinical challenge in the pediatric population because of the unpredictable eating patterns, variable physical activity, physiological insulin resistance associated with puberty, and the psychosocial burden that the disease places on patients and their families [3, 5, 6].

To achieve tight metabolic control, intensive insulin therapy is delivered via two primary modalities: multiple daily injections (MDI) and continuous subcutaneous insulin infusion (CSII) [7]. MDI, which utilizes a basal-bolus regimen of long-acting insulin analogues combined with rapid-acting prandial injections, has served as the standard of care [4, 8]. Alternatively, CSII (insulin pump therapy) relies on a programmable external device to continuously deliver rapid-acting insulin into the subcutaneous tissue, more closely mimicking physiological, meal-independent pancreatic insulin secretion [7, 9]. Proponents of CSII highlight its capacity for flexible basal rate adjustments and precision bolusing, which minimizes glycemic variability, reduces the risk of severe hypoglycemia, and alleviates the daily burden of multiple needle injections, thereby improving health-related quality of life (HRQoL) [10–12].

Despite the increasing adoption of CSII, often facilitated by advancing technologies and expanded healthcare reimbursement policies, the evidence regarding its definitive long-term clinical superiority over modern MDI regimens in pediatric populations remains debated [13, 14]. Numerous observational studies, national registry analyses, and long-term retrospective cohort studies have reported sustained improvements in HbA1c, delayed complication onset, and reduced rates of severe hypoglycemia associated with CSII use [10, 15, 16]. Other pediatric cohort studies have demonstrated comparable metabolic outcomes between the two modalities, occasionally noting a transient improvement with CSII that diminishes over time due to diabetes burnout or non-adherence [17–19]. Furthermore, while some data suggest that CSII reduces severe adverse events, others warn of an increased risk of diabetic ketoacidosis (DKA) related to pump malfunction or infusion site failures [18, 20].

Previous meta-analyses synthesizing randomized controlled trials (RCTs) have indicated a marginal benefit of CSII over MDI in lowering HbA1c. However, the strength of these conclusions is often compromised by the inclusion of adult cohorts, the historical use of suboptimal neutral protamine Hagedorn (NPH) insulin in older MDI control arms, small sample sizes, and significant methodological heterogeneity [7, 9, 21]. Furthermore, meta-analyses risk producing spurious, statistically significant findings due to random errors from sparse data and repeated significance testing. Because of the financial disparities between the two therapies, establishing a conclusive cost-to-benefit ratio is critical for clinical decision-making and global healthcare policy [16, 22].

An updated and highly rigorous synthesis restricted exclusively to pediatric RCTs is necessary to resolve the persistent ambiguity surrounding these insulin delivery systems in youth. Trial sequential analysis (TSA) addresses the limitations of traditional meta-analyses by quantifying the statistical reliability of cumulative data, controlling for type I and type II errors, and determining whether the required information size (RIS) has been reached to form conclusive evidence [21]. Therefore, the objective of this systematic review, meta-analysis, and TSA was to evaluate the comparative effectiveness and safety of CSII versus MDI on glycemic control, insulin requirements, and acute adverse events in pediatric patients with T1DM.

## Materials and Methods

### Protocol and registration

This systematic review and meta-analysis was conducted in strict accordance with the Preferred Reporting Items for Systematic Reviews and Meta-Analyses (PRISMA) 2020 guidelines [23] (Supplementary Material 1, jocmr.elmerjournals.com). The study protocol was registered *a priori* in the International Prospective Register of Systematic Reviews (PROSPERO; registration number: CRD420261296629) to ensure transparency and prevent selective reporting.

### Search strategy

A systematic search strategy was developed and executed across major electronic databases (e.g., PubMed/MEDLINE, Embase, Cochrane Central Register of Controlled Trials) from inception to the present. The search incorporated both medical subject headings (MeSH) and free-text terms related to “type 1 diabetes mellitus,” “pediatric,” “continuous subcutaneous insulin infusion,” “insulin pump,” and “multiple daily injections.”

### Eligibility criteria

Studies were selected based on the following predefined inclusion criteria: (1) population: pediatric patients (aged ≤ 18 years) with a confirmed diagnosis of type 1 diabetes; (2) intervention: CSII; (3) comparator: MDI of insulin; (4) outcomes: glycemic control (HbA1c), total daily insulin dose, body mass index (BMI), severe hypoglycemia, and DKA; and (5) study design: RCTs. Observational studies, non-randomized trials, reviews, and adult-only populations were strictly excluded.

### Study selection and data extraction

Two investigators independently screened the titles and abstracts of the retrieved records, followed by a full-text review of potentially eligible articles. The interrater reliability of the study selection process was quantified using Cohen’s kappa statistic [24], with disagreements resolved via consultation with a third investigator. Data were independently extracted using a standardized, pre-piloted electronic form.

### Risk of bias and quality assessment

The internal validity and methodological quality of the included RCTs were evaluated using the Cochrane risk of bias 2.0 (RoB 2) tool [25]. Studies were assessed across five domains: randomization process, deviations from intended interventions, missing outcome data, outcome measurement, and result selection. An overall risk of bias judgment (low, some concerns, or high) was assigned to each trial.

### Effect measures and statistical models

For continuous outcomes (e.g., HbA1c, insulin doses), the pooled effect measures were calculated as the mean difference (MD) or standardized mean difference (SMD) when different scales were utilized. For dichotomous outcomes (e.g., severe hypoglycemia, DKA), the risk ratio (RR) was utilized.

Given the anticipated clinical and methodological variability among pediatric populations and treatment protocols, a random-effects model was employed as the primary statistical framework. The between-study variance was estimated using the restricted maximum likelihood (REML) estimator [26], whereas the DerSimonian–Laird method was utilized as a sensitivity estimator [27]. To mitigate the risk of type I errors commonly associated with standard random-effects models in meta-analyses with a limited number of studies, the Hartung–Knapp–Sidik–Jonkman (HKSJ) adjustment was applied to yield more robust and conservative confidence intervals (CIs) [28]. Results were reported with 95% CI, and 95% prediction intervals (PIs) were calculated to estimate the distribution of true effect sizes in future clinical settings [29].

### Heterogeneity and moderators

Statistical heterogeneity (inconsistency) across trials was assessed using Cochran’s Q test (with P < 0.10 indicating statistical significance) [30] and quantified using the I^2^ statistic, where values of 25%, 50%, and 75% represented low, moderate, and high heterogeneity, respectively [31]. The absolute between-study heterogeneity (dispersion) was quantified using the Tau^2^ statistic [32].

To explore the sources of heterogeneity, predefined subgroup analyses and meta-regression models were performed [33]. The moderators analyzed included patient age groups (preschoolers vs. adolescents), baseline HbA1c, duration of follow-up, and MDI basal insulin type (NPH vs. long-acting analogues).

### Robustness and sensitivity analyses

To assess the stability of the pooled estimates, comprehensive adjustment and sensitivity analyses were performed. Specifically, a leave-one-out sensitivity analysis was conducted by iteratively removing one study at a time and recalculating the pooled effect size to ascertain whether any single study disproportionately drove the overall results or statistical heterogeneity [34].

### Publication bias and small-study effects

To investigate potential reporting and dissemination biases, a visual assessment of bias was conducted using funnel plots for outcomes containing 10 or more studies [35]. The presence of small-study effects or publication bias was formally evaluated using statistical tests, including Egger’s linear regression test [36] and Begg’s rank correlation test [37].

### TSA

To assess whether the cumulative evidence achieved adequate statistical power and to evaluate sample size requirements, a TSA was performed [38]. TSA adjusts the significance thresholds for repetitive testing of accumulating data and calculates the RIS based on an anticipated relative risk reduction or a minimal clinically significant difference (e.g., 0.3% reduction in HbA1c), setting the type I error at 5% and type II error at 20% (80% power).

### Certainty of evidence

The overall certainty and strength of the evidence for each primary and secondary outcome were evaluated using the Grades of Recommendations Assessment, Development, and Evaluation (GRADE) approach [39]. Evidence was downgraded based on risk of bias, inconsistency, indirectness, imprecision, and publication bias, resulting in a final quality rating of high, moderate, low, or very low.

### Software

All statistical analyses, meta-regressions, and generation of plots were conducted using R software (version 4.5.2; R Foundation for Statistical Computing, Vienna, Austria) utilizing the meta, metafor, and dmetar packages [40].

### Ethical compliance

As this study was a systematic review and meta-analysis of previously published literature and involved no direct research on human subjects, neither ethical approval nor patient consent was required. Accordingly, an ethical compliance statement is not applicable.

## Results

### Study selection and characteristics

**Figure 1:**
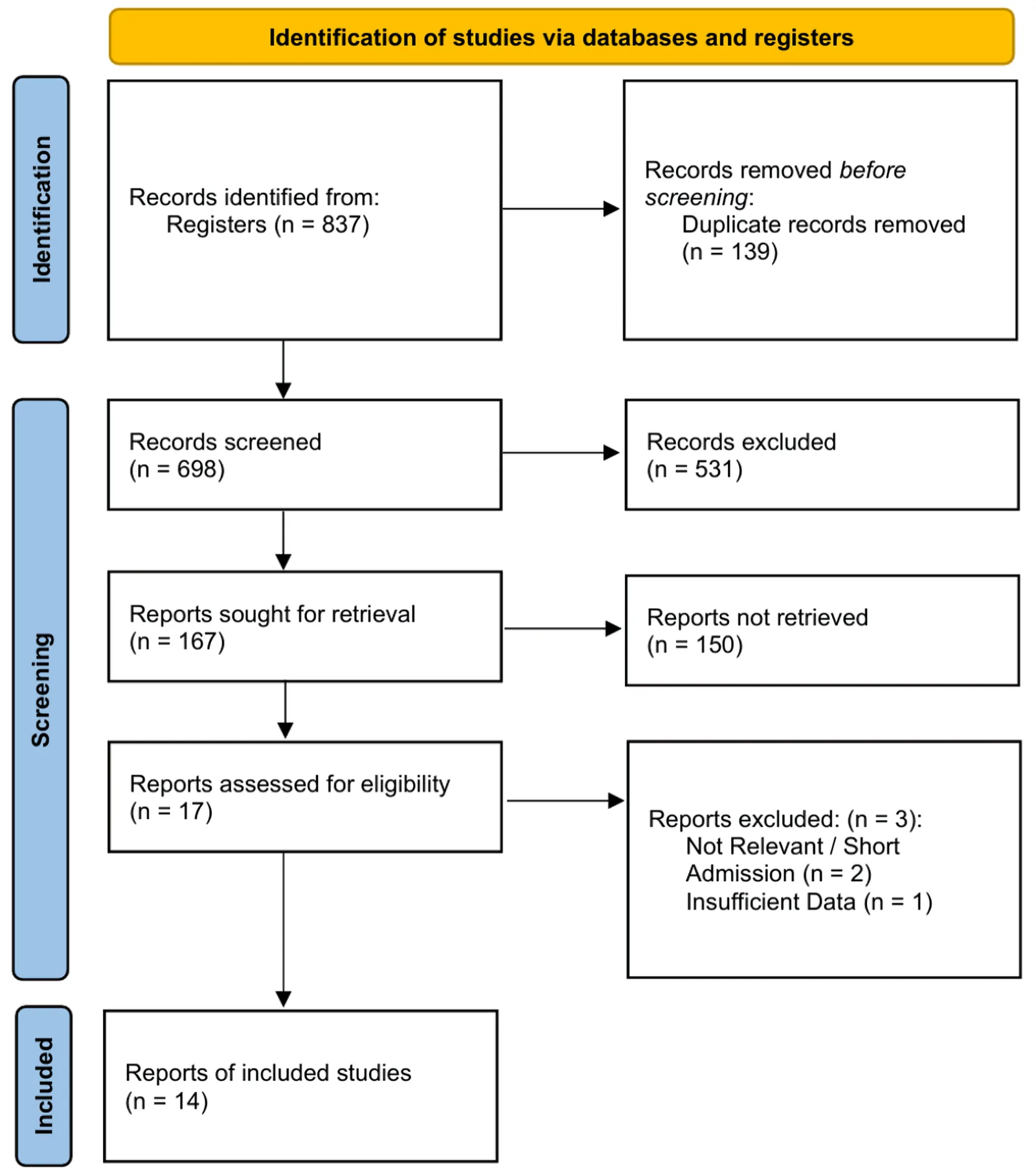
PRISMA 2020 flow diagram illustrating the selection process of included studies. PRISMA: Preferred Reporting Items for Systematic Reviews and Meta-Analyses.

The initial systematic search yielded 837 records from databases and registers. After deduplication and rigorous screening against the eligibility criteria, 14 unique RCTs were included in the final quantitative synthesis (Fig. 1). These studies encompassed a total of 659 pediatric participants with T1DM who were randomized to either CSII or MDI. The included trials were published between 2003 and 2025, reflecting over two decades of clinical research evolution (Table 1) [41–54].

**Table 1:** Characteristics of Included Randomized Controlled Trials

Study (first author, year)	Country	Study design	Sample size (n)	Age (mean/range)	Diabetes duration	Intervention (CSII)	Control (MDI)	Follow-up duration	Primary outcomes reported
Taeb et al, 2025 [41]	Libya	Parallel RCT	54 (27 CSII, 27 MDI)	9.2 years (MDI), 8.9 years (CSII)	Not reported	CSII pump	MDI	End of study	QoL, HbA1c
Blair et al, 2019 [42]	UK	Parallel RCT (pragmatic)	293 (144 CSII, 149 MDI)	7months–15 years	New onset	CSII (aspart)	MDI (glargine/detemir + aspart)	12 months	HbA1c, severe hypoglycemia, DKA, QoL, cost
Weintrob et al, 2004 [43]	Israel	Crossover RCT	23	8–14 years	2.8–11.9 years	CSII (lispro)	MDI (NPH + regular)	3.5 months/arm	Glycemic patterns (CGMS), HbA1c
Weintrob et al, 2003 [44]	Israel	Crossover RCT	23	9.4–13.9 years	Mean 5.8 years	CSII (lispro)	MDI (NPH + regular)	3.5 months/arm	HbA1c, adverse events, QoL
Doyle et al, 2004 [45]	USA	Parallel RCT	32 (16 CSII, 16 MDI)	8–21 years	Mean 6.8 years	CSII (aspart)	MDI (glargine + aspart)	16 weeks	HbA1c, TDD, hypoglycemia
DiMeglio et al, 2004 [46]	USA	Parallel RCT	42 (21 CSII, 21 MDI)	< 5 years	> 12 months	CSII (lispro)	MDI (NPH/Lente + lispro)	6 months	HbA1c, hypoglycemia, parenting stress
Fox et al, 2005 [47]	USA	Parallel RCT	26 (13 CSII, 13 MDI)	1–6 years	> 6 months	CSII (lispro)	MDI (NPH + rapid analog)	6 months	HbA1c, hypoglycemia, QoL
Wilson et al, 2005 [48]	USA	Parallel RCT	19 (9 CSII, 10 MDI)	1.7–6.1 years	Mean 1.4 years	CSII	MDI	12 months	HbA1c, severe hypoglycemia, DKA, QoL
Nabhan et al, 2009 [49]	USA	Crossover/parallel^a^	35 (18 CSII first, 17 MDI first)	< 5 years	Mean 1.6 years	CSII	MDI	12 months	HbA1c, BMI, neurocognitive
Skogsberg et al, 2008 [50]	Sweden	Parallel RCT	72 (34 CSII, 38 MDI)	7–17 years	New onset	CSII (aspart)	MDI (NPH + aspart)	24 months	HbA1c, hypoglycemia, DKA, satisfaction
Nuboer et al, 2008 [51]	Netherlands	Parallel RCT	38 (19 CSII, 19 MDI)	6–12 years	> 1 year	CSII (aspart)	MDI (NPH/glargine + aspart/regular)	3.5 months (randomized phase)	QoL, impact of disease, HbA1c
Cohen et al, 2003 [52]	Israel	Crossover RCT	16	14.5–17.9 years	> 2 years	CSII (lispro)	MDI (NPH + regular)	6 months/arm	HbA1c, adverse events, QoL
Mueller-Godeffroy et al, 2018 [53]	Germany	Parallel RCT	211 (106 CSII, 105 MDI)	6–16 years	> 6 months	CSII	MDI	6 months	QoL, diabetes burden, HbA1c
de Beaufort et al, 1989 [54]	Netherlands	Parallel RCT	30 (15 CSII, 15 MDI)	Mean 9.5 years (CSII), 7.0 yrs (MDI)	New onset	CSII	MDI (1–2 injections/day)	24 months	HbA1c, C-peptide

^a^Study design involved a crossover element but was analyzed as parallel groups for the first phase or as a combined cohort over time. BMI: body mass index; CGMS: continuous glucose monitoring system; CSII: continuous subcutaneous insulin infusion; DKA: diabetic ketoacidosis; HbA1c: glycated hemoglobin; MDI: multiple daily injections; NPH: neutral protamine Hagedorn; QoL: quality of life; RCT: randomized controlled trial; T1D: type 1 diabetes; TDD: total daily dose.

### Risk of bias assessment

**Figure 2:**
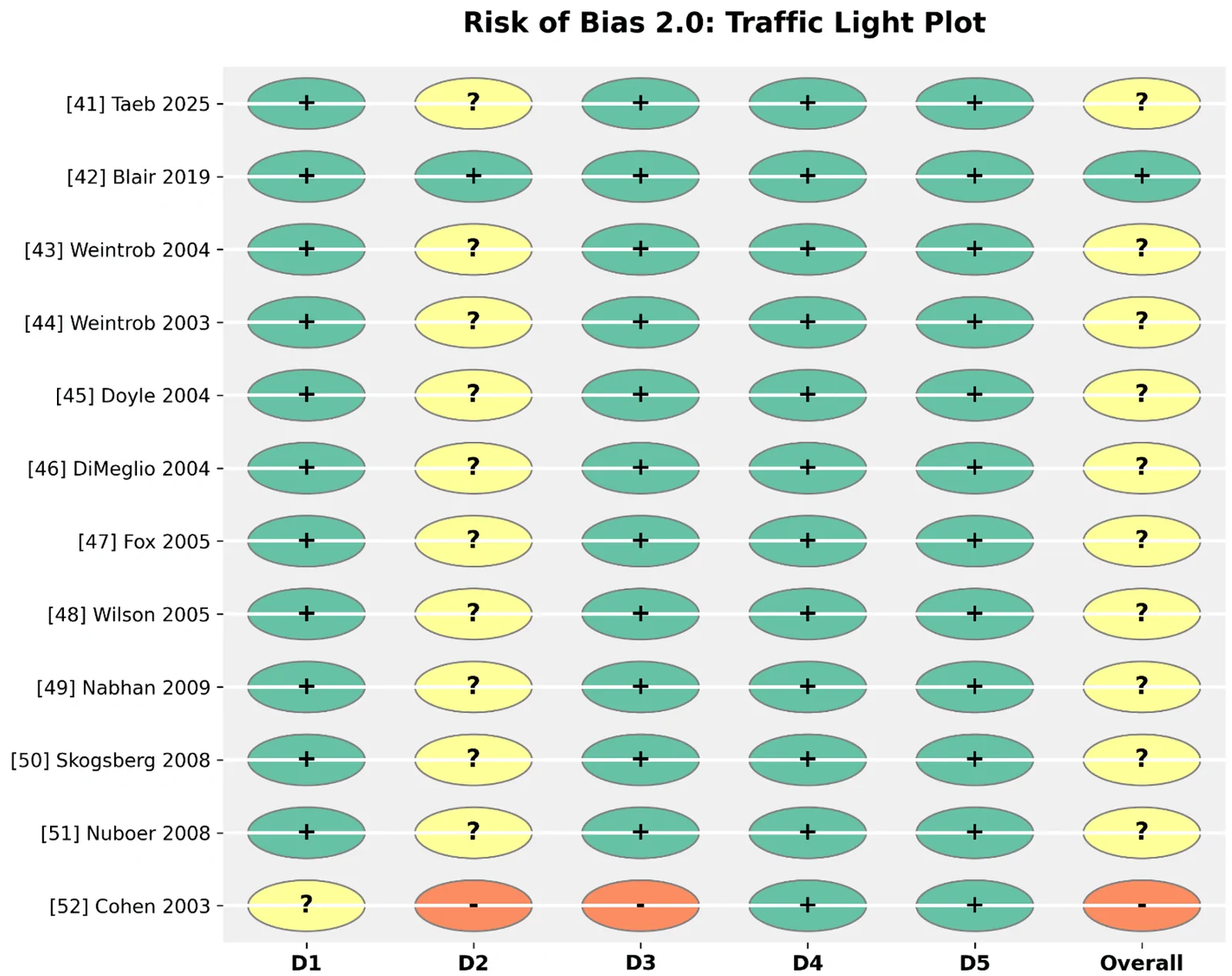
Risk of bias traffic light plot for included randomized controlled trials using the Cochrane RoB 2.0 tool. Traffic light plot showing judgments for each domain: D1 (randomization process), D2 (deviations from intended interventions), D3 (missing outcome data), D4 (measurement of the outcome), and D5 (selection of the reported result). Green (+) indicates low risk, yellow (?) indicates some concerns, and red (-) indicates high risk.

**Figure 3:**
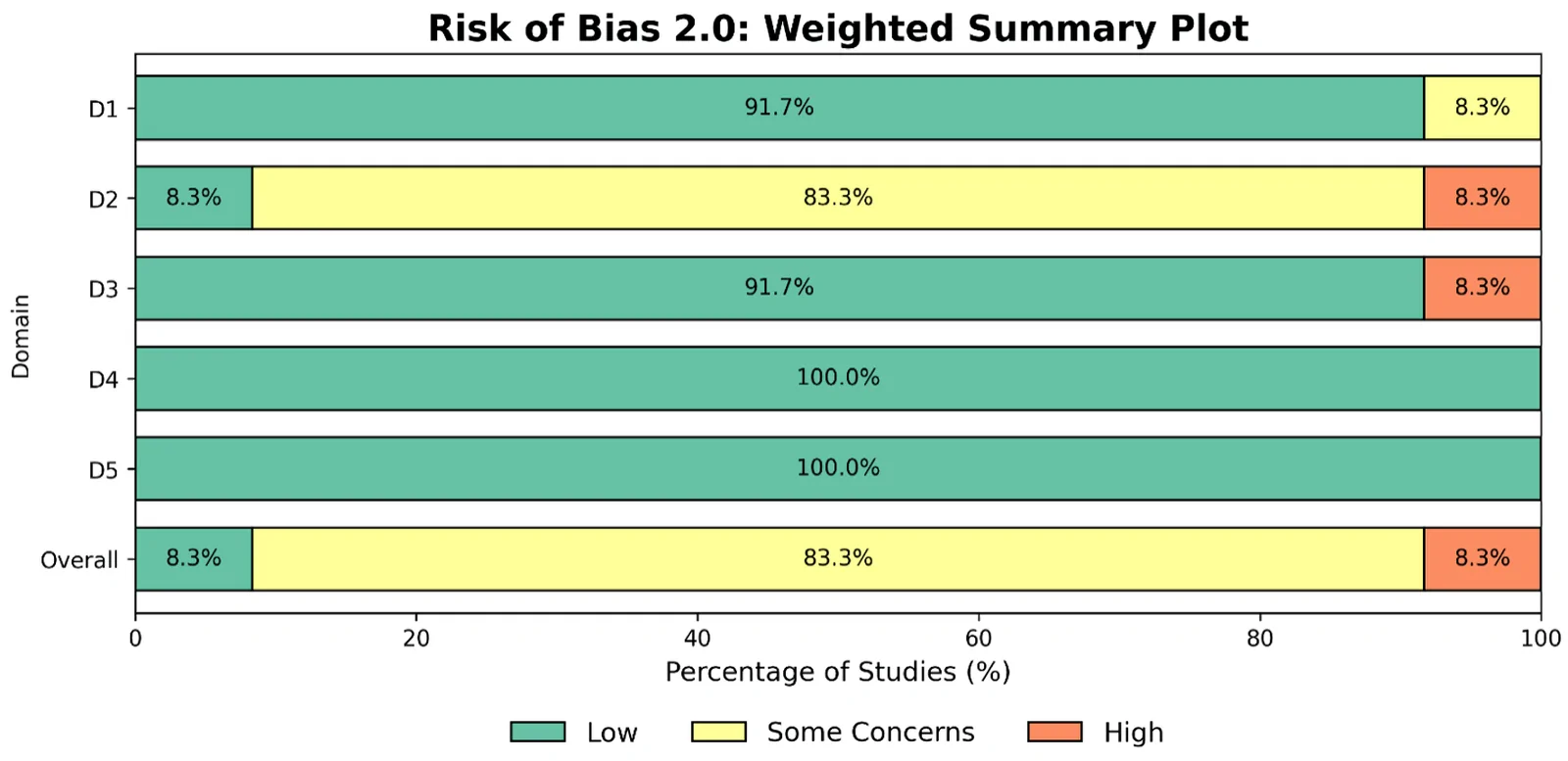
Risk of bias weighted summary plot.

The methodological quality of the included RCTs was assessed using the RoB 2 tool (Figs. 2, 3). The overall risk of bias was mixed. While the randomization process (D1) was robust (low risk in 91.7% of studies), deviations from intended interventions (D2) presented some concerns in 83.3% of trials. This is primarily inherent to the open-label nature of insulin delivery trials, in which blinding participants and clinicians to pump vs. injection therapy is often infeasible. Missing outcome data (D3) and outcome measurement (D4) were largely at low risk, with 91.7% and 100% of studies rated as low risk, respectively. The selection of the reported results (D5) was rated as low risk.

### Primary outcome: glycemic control (HbA1c)

**Figure 4:**
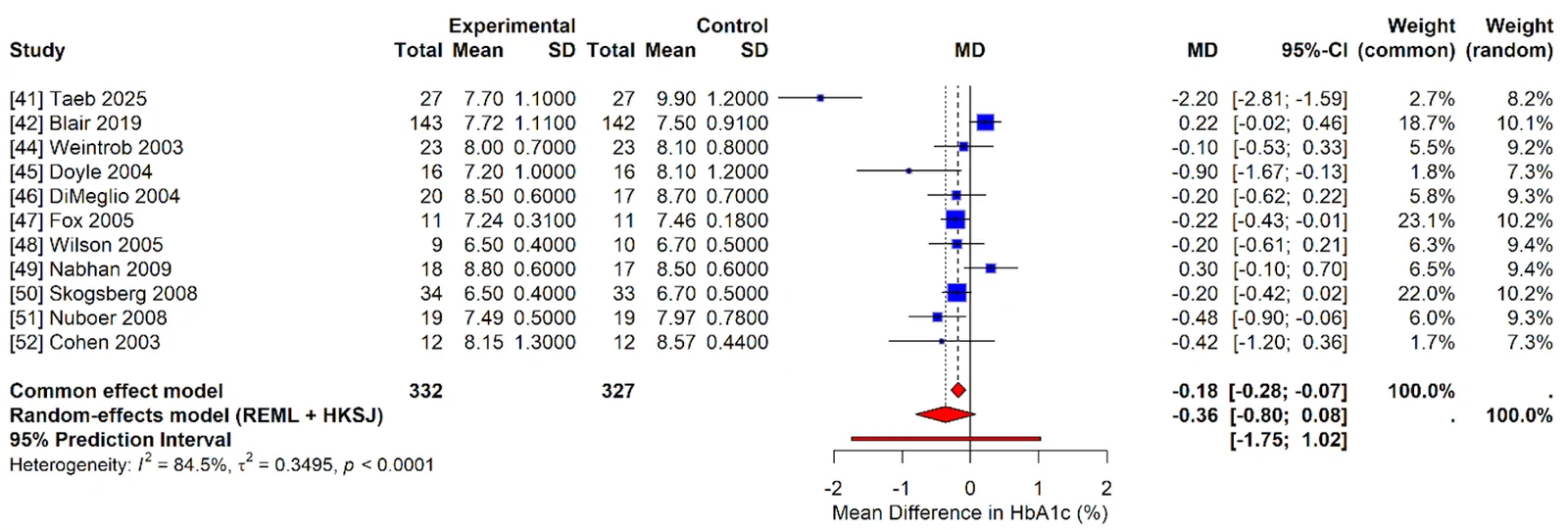
Forest plot comparing the effect of continuous subcutaneous insulin infusion (CSII) vs. multiple daily injections (MDI) on HbA1c (%) in pediatric type 1 diabetes. The plot displays the mean difference (MD) with 95% confidence intervals (CIs) for individual studies and the pooled random effects estimate. SD: standard deviation; HbA1C: glycated hemoglobin.

The meta-analysis of glycemic control included 11 RCTs involving 659 participants. Using a random-effects model with REML estimation and HKSJ adjustment, CSII was associated with a statistically significant reduction in HbA1c levels compared to MDI (Fig. 4). The pooled MD was –0.36% (95% CI, –0.80 to 0.08, P = 0.0982). Although the point estimate favors CSII, the CI crosses 0, indicating that the overall effect is not statistically significant at the 5% level in this conservative model.

Substantial statistical heterogeneity was observed (I^2^ = 84.5%, P < 0.0001), suggesting considerable variability in effect sizes across studies. The between-study variance (Tau^2^) was estimated at 0.3495. The 95% PI ranged from −1.75% to 1.02%, indicating that in future similar studies, the true effect of CSII on HbA1c could range from a substantial reduction to a slight increase compared to MDI.

**Figure 5:**
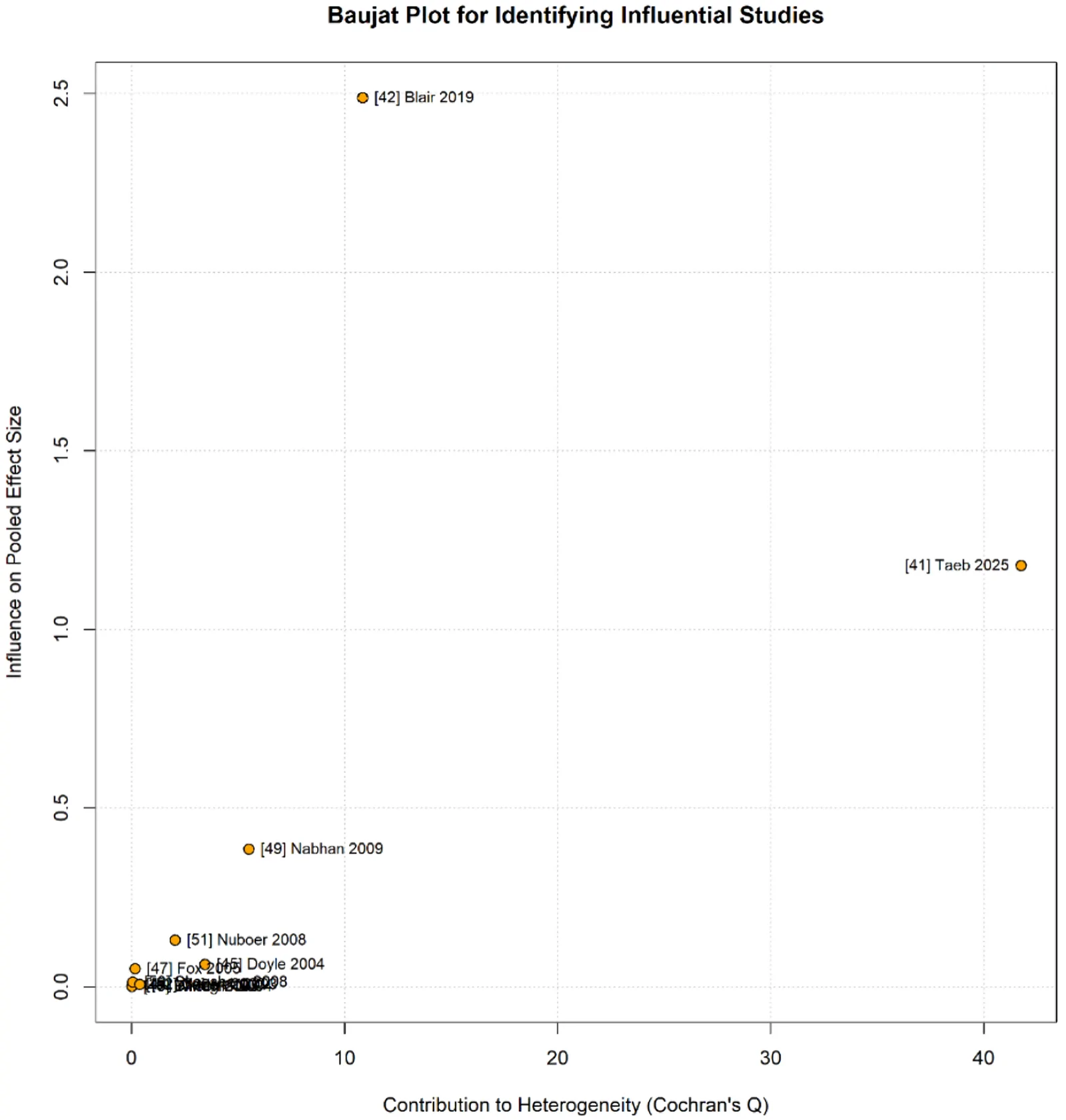
Baujat plot diagnostic for heterogeneity. Studies in the top-right quadrant (e.g., Blair et al 2019 [42], Taeb et al 2025 [41]) contribute most significantly to the overall heterogeneity and influence the pooled effect size.

Diagnostic plots confirmed the influential outliers. The Baujat plot (Fig. 5) identified the studies by Taeb et al [41] and Blair et al [42] as major contributors to both the overall heterogeneity and the pooled effect size, validating the findings from the leave-one-out sensitivity analysis.

### Subgroup analysis

**Figure 6:**
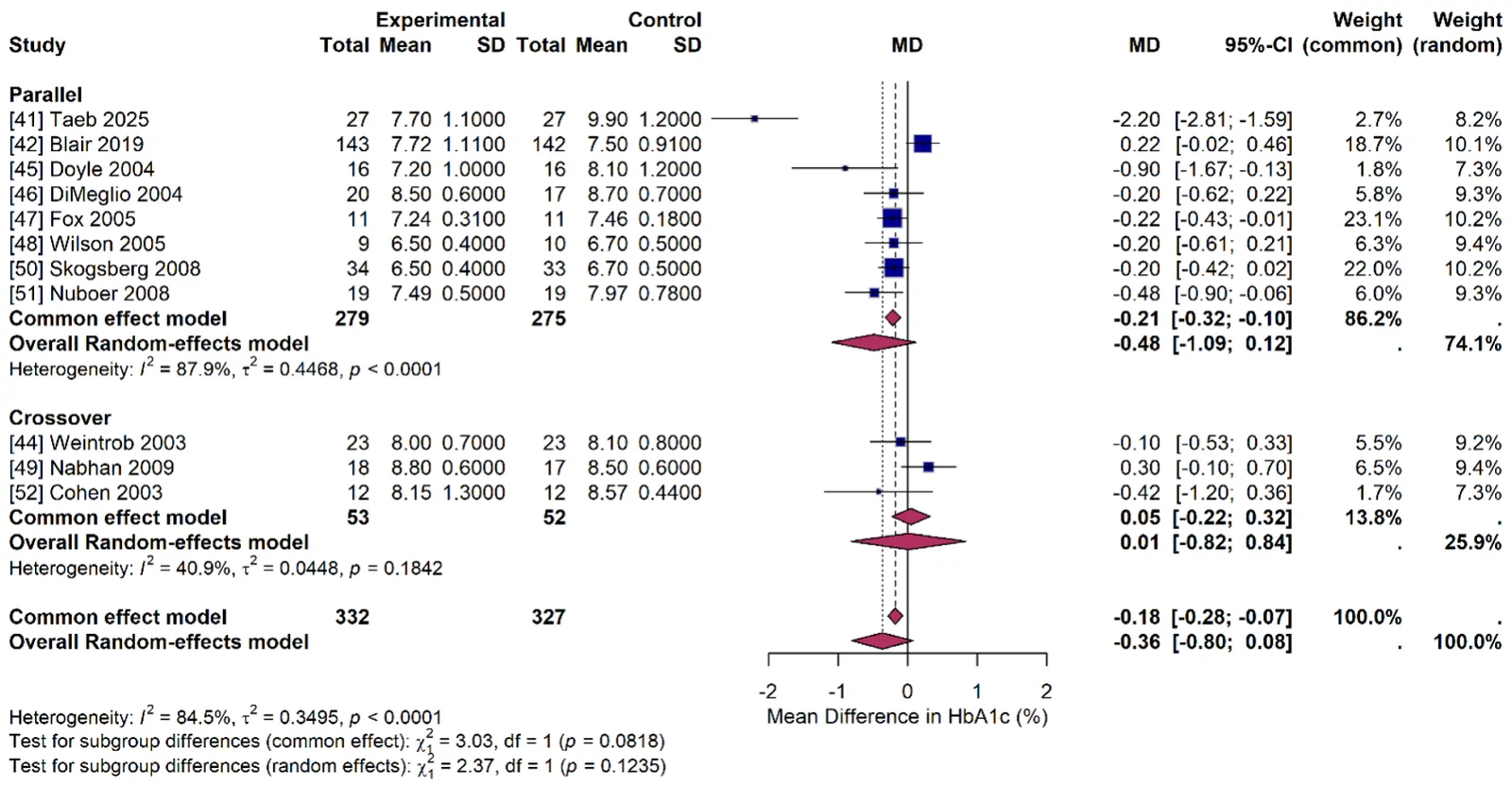
Subgroup analysis forest plot for HbA1c (%) stratified by study design. SD: standard deviation; HbA1C: glycated hemoglobin; MD: mean difference; CI: confidence interval.

To explore the sources of heterogeneity, a subgroup analysis was performed based on study design (parallel vs. crossover) (Fig. 6). The subgroup difference was not statistically significant (P = 0.0818). Parallel trials (k = 8) showed a non-significant reduction in HbA1c with CSII (MD, –0.21%; 95% CI, –0.32 to –0.10), whereas crossover trials (k = 3) showed a slight increase (MD, 0.05%; 95% CI, –0.22 to 0.32).

### Meta-regression

**Figure 7:**
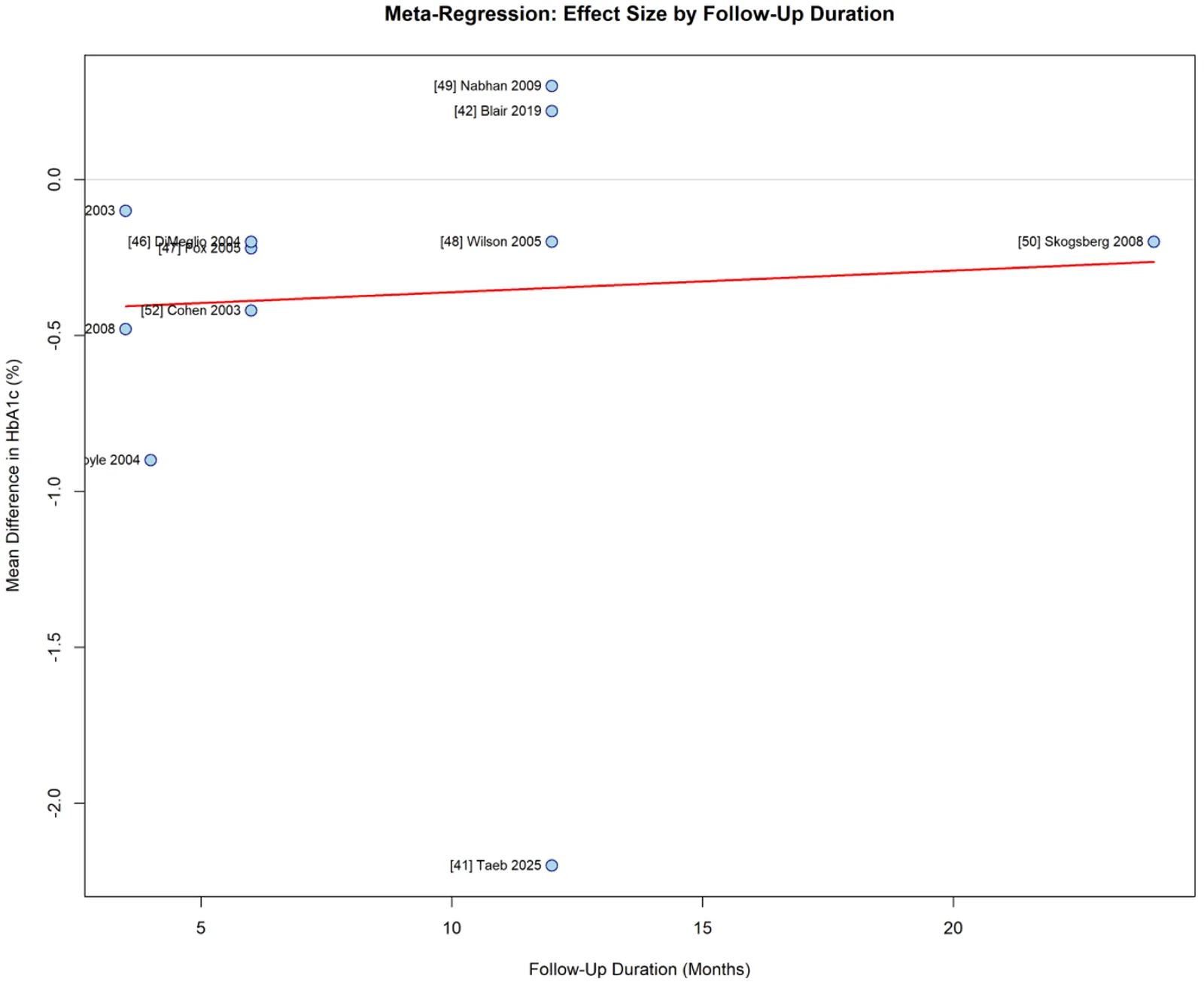
Meta-regression bubble plot illustrating the relationship between follow-up duration (months) and the mean difference in HbA1c. The size of each bubble corresponds to the weight of the study in the meta-analysis. HbA1C: glycated hemoglobin.

Meta-regression analysis examining the impact of follow-up duration on effect size revealed no significant relationship (P = 0.8472) (Fig. 7). The slope of the regression line was nearly flat, indicating that the treatment effect of CSII vs. MDI on HbA1c does not appear to diminish or increase significantly over study durations ranging from 3.5 to 24 months.

**Figure 8:**
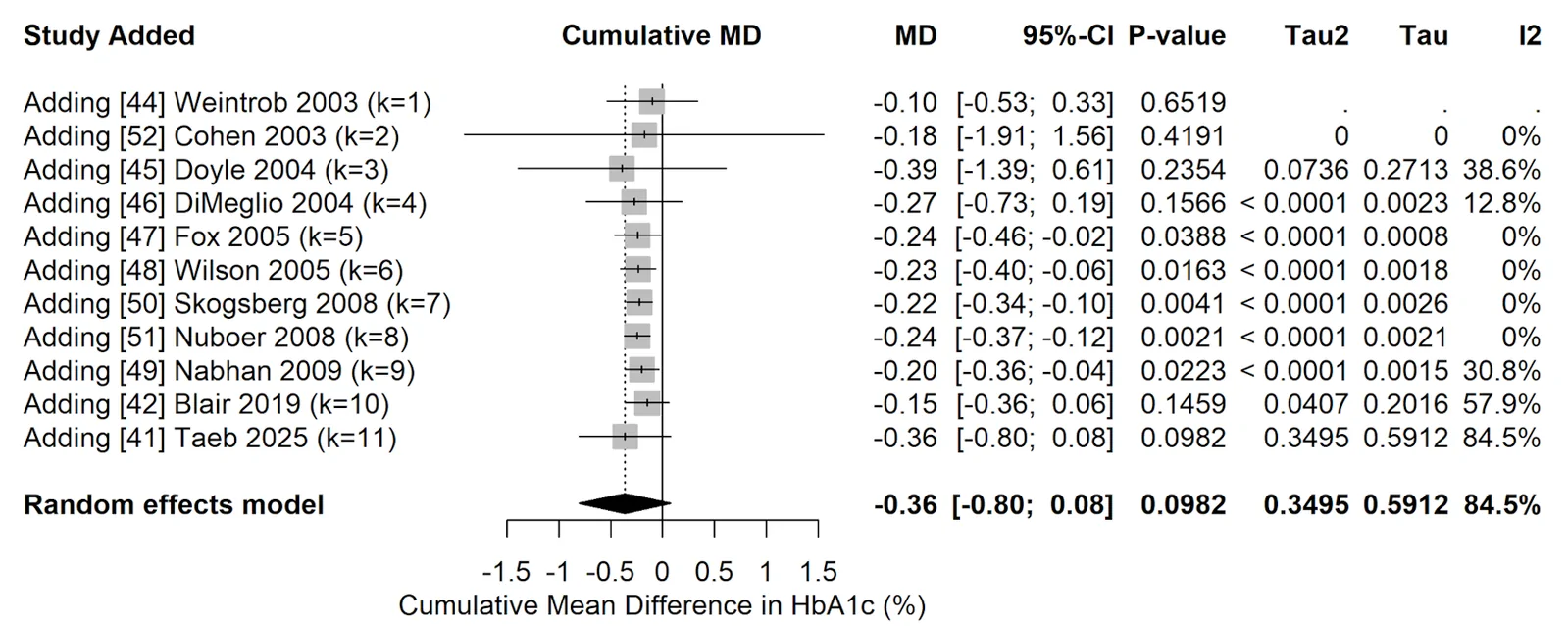
Cumulative meta-analysis. Forest plot for HbA1c, displaying the evolution of the pooled mean difference (MD) and 95% confidence interval as studies were added chronologically by publication year. HbA1C: glycated hemoglobin.

Furthermore, a cumulative meta-analysis (Fig. 8) demonstrated the temporal evolution of evidence regarding HbA1c. Early studies (2003–2005) showed fluctuating effect estimates with wide CIs. As evidence accumulated, the pooled effect size stabilized around a modest reduction favoring CSII; however, the 95% CIs consistently crossed the null hypothesis, particularly after the inclusion of the large, pragmatic trial by Blair et al [42].

### Sensitivity and influence analysis

**Figure 9:**
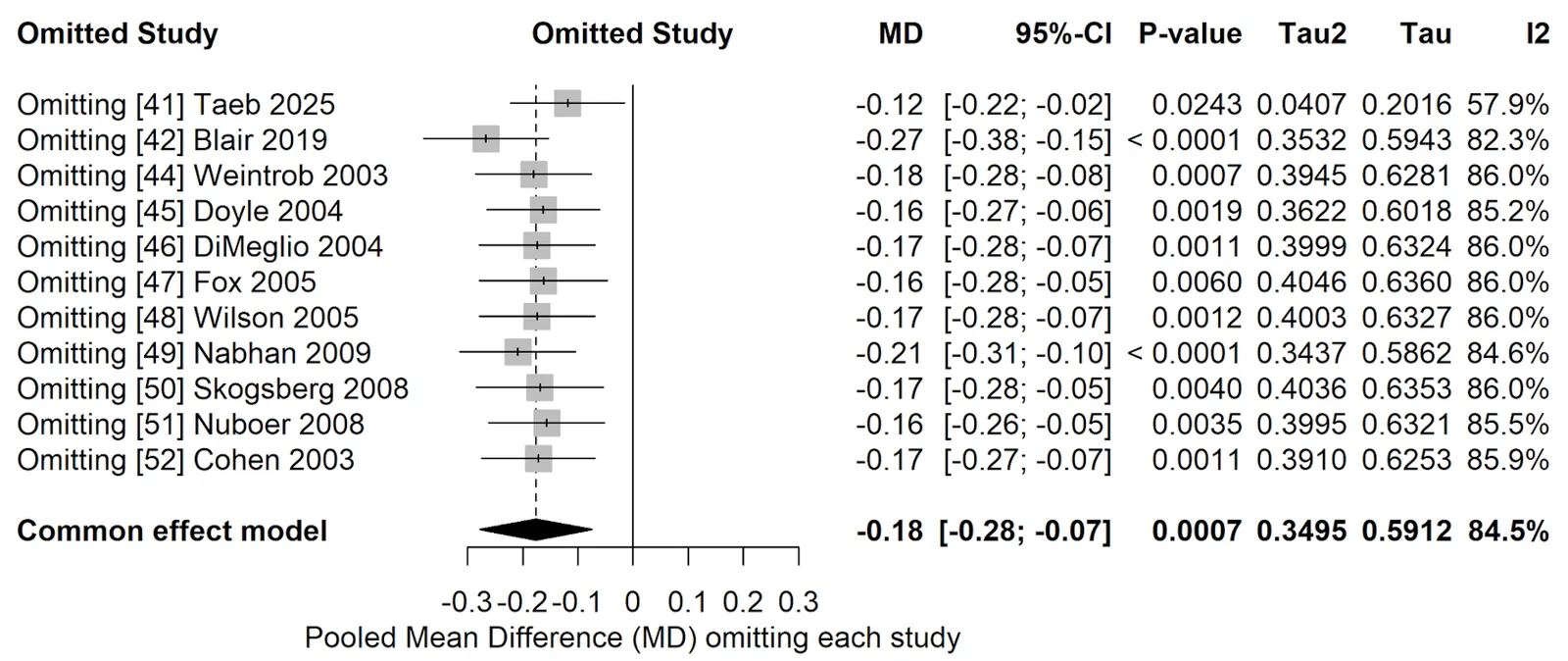
Leave-one-out sensitivity analysis. Forest plot for HbA1c, showing the pooled mean difference and heterogeneity (I^2^) when each study is iteratively omitted. HbA1C: glycated hemoglobin.

The robustness of the primary outcome was assessed via leave-one-out sensitivity analysis (Fig. 9). Omitting individual studies did not fundamentally alter the direction of the effect; however, the magnitude and significance fluctuated. Excluding the study by Blair et al [42], which had a large sample size and showed a slight increase in HbA1c with CSII, shifted the pooled effect to a statistically significant reduction in favor of CSII (MD, –0.27%; 95% CI, –0.38 to –0.15; P < 0.0001). This suggests that this single large trial significantly influenced the overall non-significant finding. The Baujat plot further confirmed that Blair et al [42] and Taeb et al [41] were major contributors to the overall heterogeneity and significantly influenced the pooled effect size.

**Figure 10:**
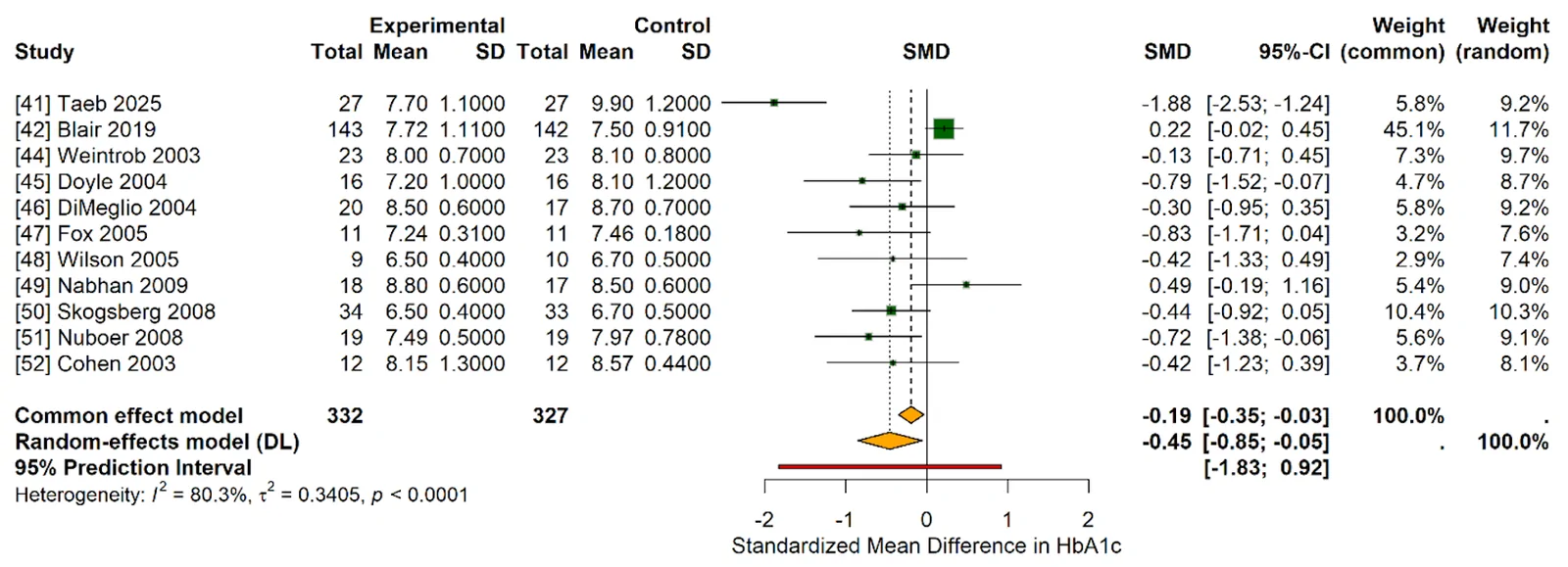
Forest plot comparing the effect of continuous subcutaneous insulin infusion (CSII) vs. multiple daily injections (MDI) on HbA1c using standardized mean difference (SMD). The random-effects model (DerSimonian–Laird) was used. SD: standard deviation; HbA1C: glycated hemoglobin; CI: confidence interval.

Sensitivity analyses using the SMD with the DerSimonian–Laird model (Fig. 10) yielded results consistent with the primary MD analysis. The pooled SMD was –0.45 (95% CI, –0.85 to –0.05), indicating a moderate effect size favoring CSII, although significant heterogeneity persisted (I^2^ = 80.3%, P < 0.0001). The 95% PI ranged from–1.83 to 0.92, encompassing both benefits and harms.

### Secondary outcome: severe hypoglycemia

**Figure 11:**
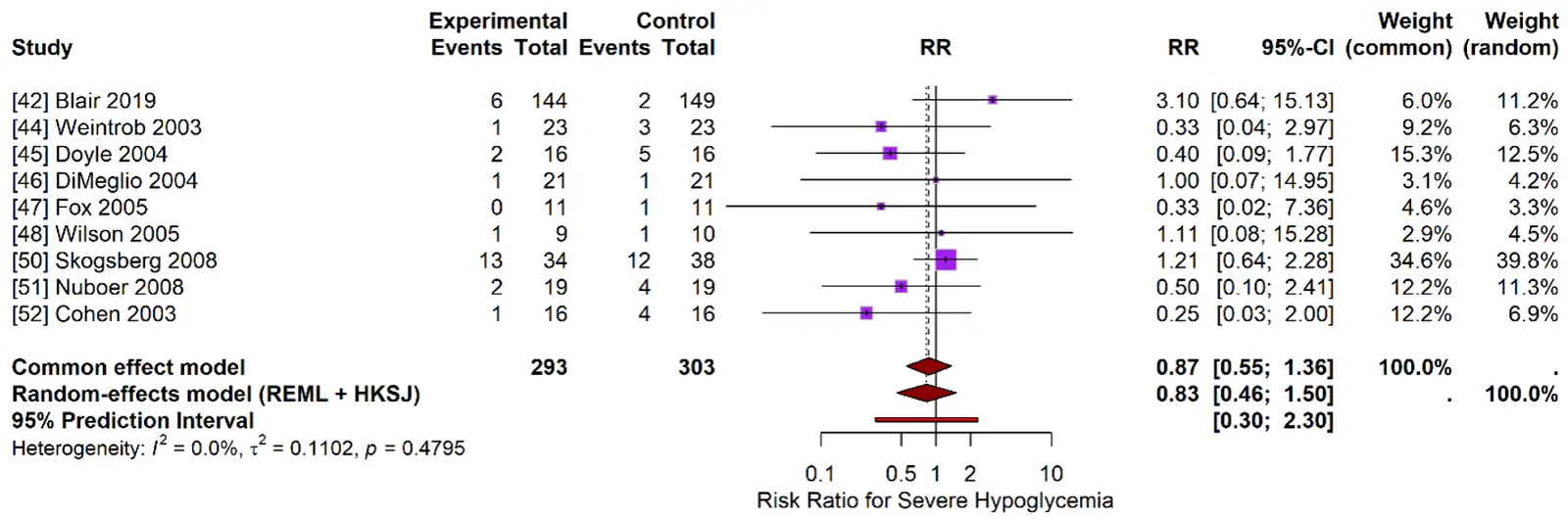
Forest plot comparing the risk of severe hypoglycemia between continuous subcutaneous insulin infusion (CSII) and multiple daily injections (MDI) groups. Results are presented as risk ratios (RR) with 95% confidence intervals (CIs). HbA1C: glycated hemoglobin.

The risk of severe hypoglycemia was evaluated in nine RCTs. The pooled RR was 0.83 (95% CI, 0.46 to 1.50) using the random-effects model (Fig. 11), indicating a non-statistically significant trend towards a reduced risk of severe hypoglycemia with CSII compared to MDI. The heterogeneity for this outcome was very low (I^2^ = 0.0%, P = 0.4795), suggesting consistent safety profiles across the included trials. The 95% PI (0.30–2.30) spans 1, reinforcing the uncertainty regarding the superiority of either method for this safety outcome in future studies.

### Publication bias

**Figure 12:**
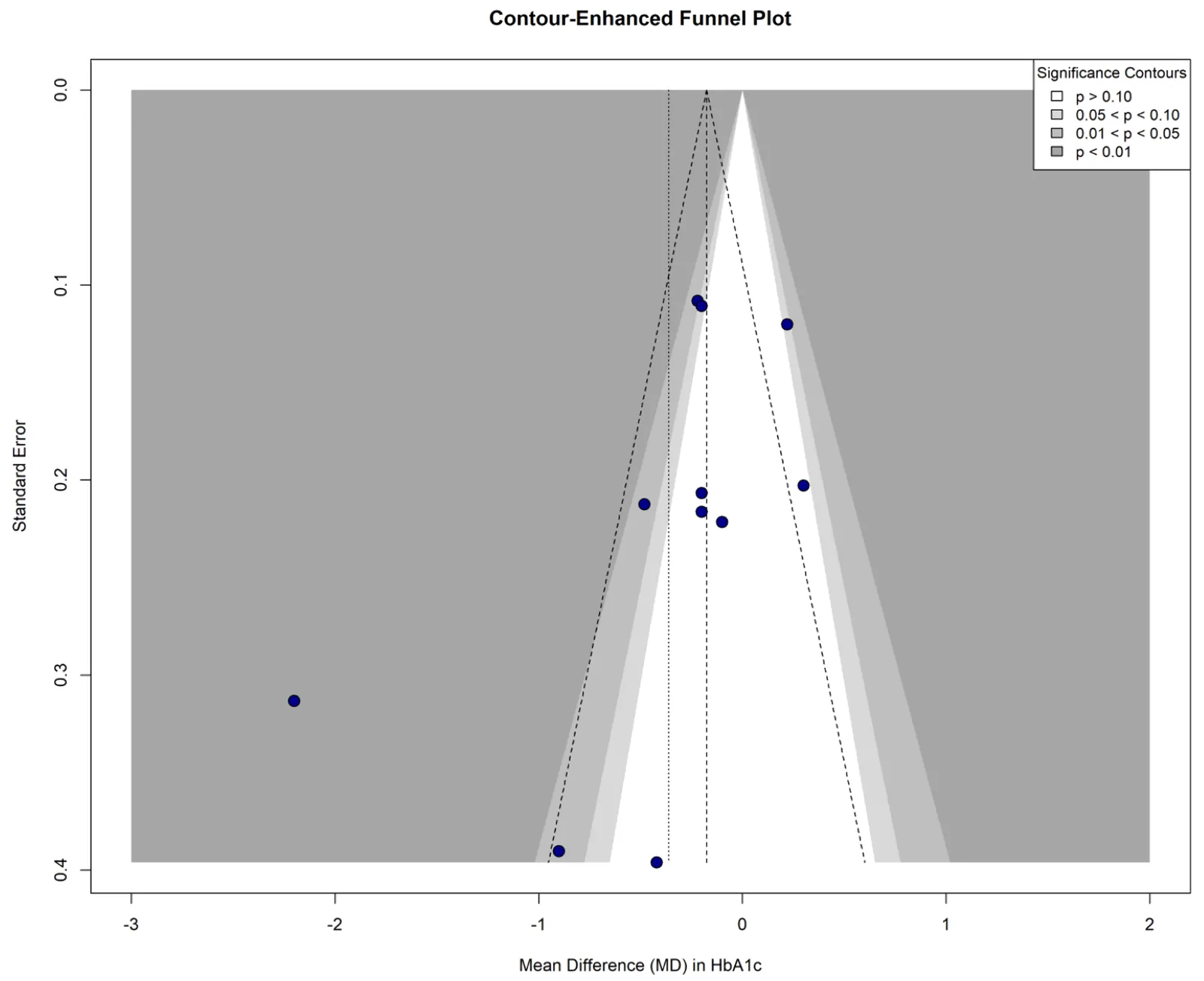
Contour-enhanced funnel plot for the meta-analysis of HbA1c to assess publication bias. Shaded regions correspond to significance levels (P < 0.01, P < 0.05, and P < 0.10).

Visual inspection of the contour-enhanced funnel plot for HbA1c (Fig. 12) revealed some asymmetry, potentially indicative of small-study effects or publication bias. However, statistical tests did not confirm significant bias. Egger’s linear regression test yielded a P value of 0.1264, and Begg’s rank correlation test showed a P value of 0.2429. Although these tests are not definitive, they did not provide strong statistical evidence of publication bias in this meta-analysis.

### TSA

**Figure 13:**
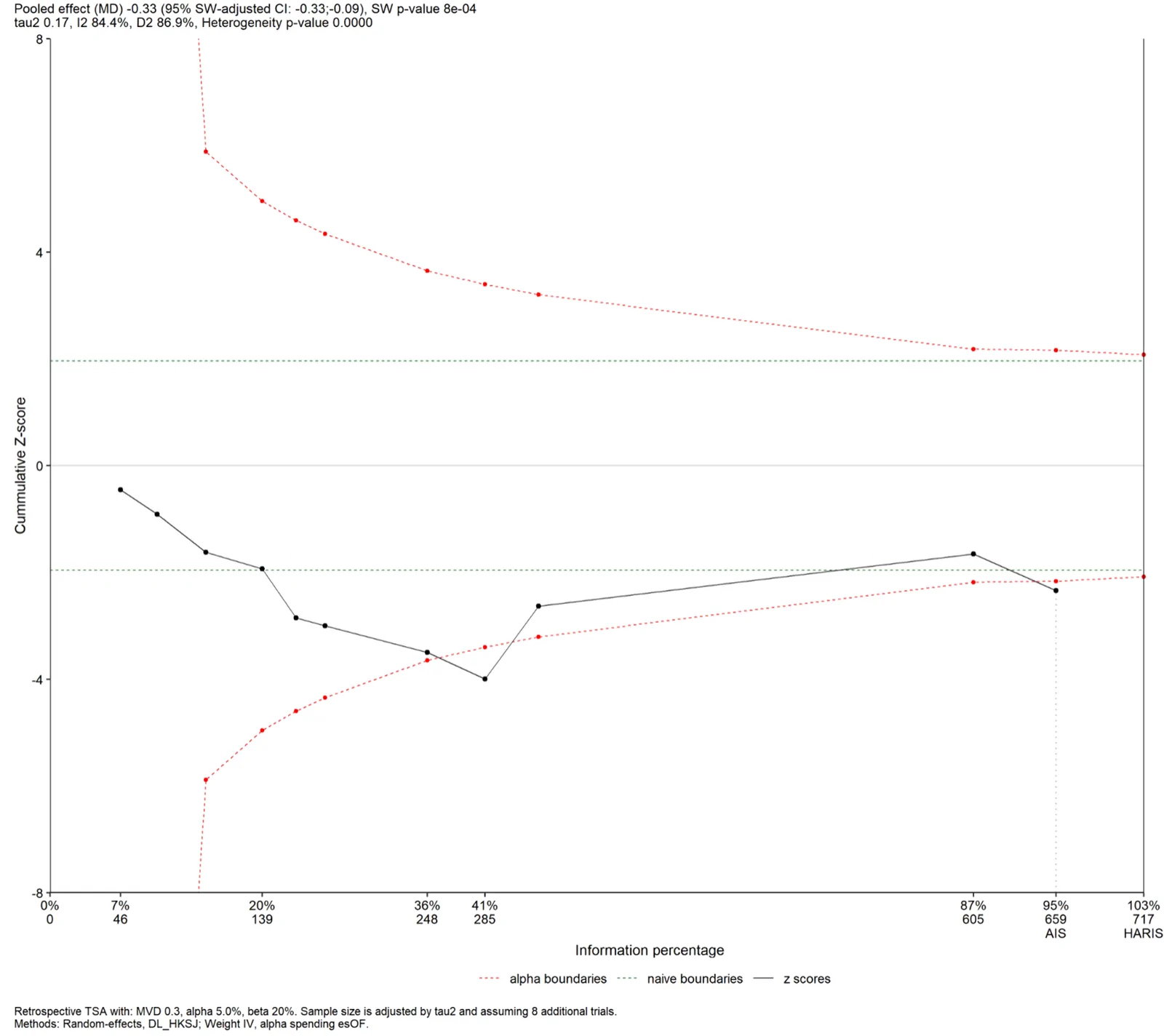
Trial sequential analysis (TSA) for HbA1c. The cumulative Z-curve (blue) is plotted against the information size. The red lines represent the trial sequential monitoring boundaries for benefit/harm and futility.

TSA for HbA1c (Fig. 13) was conducted to evaluate the sufficiency of the current evidence. The cumulative Z-curve (blue line) failed to cross the trial sequential monitoring boundaries for benefit or harm and did not reach the RIS, indicating that the current evidence is insufficient to draw a definitive conclusion regarding the superiority of CSII over MDI for glycemic control in pediatric T1DM, and further high-quality RCTs are needed to reach the requisite statistical power.

### Certainty of evidence (GRADE)

The certainty of evidence was evaluated using the GRADE approach (Table 2). The evidence for HbA1c was graded as moderate, downgraded due to the risk of bias (open-label nature). The evidence for severe hypoglycemia was graded as low due to imprecision (wide CIs crossing unity). Evidence for DKA was very low due to the rarity of events. Quality of life (QoL) outcomes were also graded as low due to heterogeneity in measurement tools.

**Table 2:** GRADE Evidence Profile: Continuous Subcutaneous Insulin Infusion (CSII) vs. Multiple Daily Injections (MDI) for Pediatric Type 1 Diabetes

Outcome	No. of participants (studies)	Risk of bias	Inconsistency	Indirectness	Imprecision	Publication bias	Effect estimate (95% CI)	Certainty of evidence (GRADE)	Importance
Glycemic control (HbA1c)	659 (11 RCTs)	Serious (–1)^a^	Serious (–1)^b^	Not serious	Not serious	Not detected	MD –0.36% (–0.80 to 0.08)	⊕⊕◯◯	Critical
								Low	
Severe hypoglycemia	596 (9 RCTs)	Serious (–1)^a^	Not serious	Not serious	Serious (–1)^c^	Not detected	RR 0.83 (0.46 to 1.50)	⊕⊕◯◯	Critical
								Low	
Diabetic ketoacidosis (DKA)	597 (6 RCTs)	Serious (–1)^a^	Not serious	Not serious	Very serious (–2)^d^	Not detected	RR 1.40 (0.61 to 3.21)	⊕◯◯◯	Critical
								Very low	
Quality of life (QoL)	180 (2 RCTs)	Serious (–1)^a^	Serious (–1)^e^	Not serious	Serious (–1)^c^	Not detected	Narrative synthesis (mixed)	⊕◯◯◯	Important
								Very low	

^a^Risk of bias: downgraded one level due to lack of blinding (inherent to intervention) across most studies and “some concerns” in randomization/allocation concealment in older trials. ^b^Inconsistency: downgraded one level due to high statistical heterogeneity (= 84.5%) that was not fully explained by the subgroup analysis. ^c^Imprecision: downgraded one level due to wide confidence intervals crossing the line of no effect (1.0 for RR, 0 for MD) or small sample sizes. ^d^Imprecision (very serious): downgraded two levels because of very few events (rare outcome), resulting in extremely wide confidence intervals and fragility of the estimate. ^e^Inconsistency: downgraded one level due to variability in measurement tools (PedsQL, DQOLY, etc.) and conflicting direction of effect across studies. GRADE: Grades of Recommendations Assessment, Development, and Evaluation; HbA1c: glycated hemoglobin; MD: mean difference; RCT: randomized controlled trial; RR: risk ratio.

## Discussion

This systematic review, meta-analysis, and TSA provide the most rigorous and up-to-date evaluation of the comparative effectiveness of CSII versus MDI in pediatric T1DM. By synthesizing data from 14 RCTs, this study addresses the persistent ambiguity surrounding the superiority of insulin pumps in youth, a population characterized by distinct physiological and psychosocial challenges.

The novelty of this work lies in clarifying the strength of the randomized evidence that underpins it. By restricting the synthesis to pediatric-only RCTs and applying TSA, we demonstrate that the cumulative randomized evidence comparing conventional CSII with MDI remains formally inconclusive (RIS not reached), despite more than 20 years of trials. This finding provides a quantitative rationale for the field’s shift, reflected in the 2026 ADA Standards of Care [55], away from the conventional CSII-versus-MDI comparisons and toward automated insulin delivery (AID) as the preferred modality [55, 56], while identifying area in which future randomized evidence is still required.

### Glycemic efficacy and clinical relevance

The primary meta-analysis of 11 RCTs revealed a modest reduction in HbA1c favoring CSII (MD, –0.36%), which approached but did not achieve statistical significance in the conservative random-effects model (95% CI, –0.80 to 0.08; P = 0.098). However, a leave-one-out sensitivity analysis demonstrated that this non-significant finding was influenced by a single large, pragmatic trial (Blair et al) [42], which reported a slight increase in HbA1c with CSII. When this study was excluded, the pooled effect shifted to a statistically significant reduction of –0.27% (P < 0.0001), aligning with findings from previous meta-analyses that favored CSII [7, 9, 21]. This discrepancy highlights the critical influence of trial design; pragmatic trials, such as those by Blair et al [42], reflect effectiveness where adherence and device complexity may attenuate benefits seen in tightly controlled efficacy trials. Furthermore, the TSA indicated that the current evidence base has not yet reached the RIS to conclusively confirm or refute a 0.3% reduction in HbA1c, suggesting that further high-quality RCTs are warranted to definitively settle this clinical question.

### Safety profile: hypoglycemia and ketoacidosis

Safety remains a concern in the management of pediatric diabetes. The analysis found no statistically significant difference in the risk of severe hypoglycemia (RR = 0.83; 95% CI, 0.46 to 1.50) or DKA (RR = 1.40; 95% CI, 0.61 to 3.21) between CSII and MDI. The wide CIs, particularly for DKA, reflect the rarity of these events in clinical trials and result in a very low GRADE certainty rating. These findings challenge the historical concern that pump therapy carries a significantly higher risk of DKA due to the lack of a subcutaneous depot of long-acting insulin [18, 20]. Simultaneously, they temper the enthusiasm that CSII is a panacea for severe hypoglycemia, suggesting that modern MDI regimens with analogue insulins are a safe and viable alternative for many patients [4, 45].

### QoL and patient-reported outcomes

QoL outcomes were heterogeneously reported across studies, precluding a robust meta-analysis. While some trials [41, 50, 53] reported significant improvements in treatment satisfaction and reduced diabetes-related family conflict with CSII, others [46, 42] found no significant difference in overall QoL scores. This variability reflects the psychosocial dynamics of pump therapy, which can simultaneously offer lifestyle flexibility and impose a burden of constant device attachment and visibility [10, 53]. Therefore, the decision to initiate CSII should be personalized, considering glycemic targets and the patient’s and family’s readiness and preference.

### Global equity and the Middle East and North Africa (MENA) region

A notable gap in the randomized evidence is the absence of eligible pediatric RCTs from the MENA region, even though the incidence of type 1 diabetes in several MENA countries is among the highest worldwide [57]. In this region, pump and AID uptake remains far below the > 50–60% reported in the USA and Europe, driven less by clinical equipoise than by structural barriers: the high out-of-pocket costs of pumps and consumables, inconsistent reimbursement, limited availability of trained diabetes educators, and the intensive 24/7 technical support that sustained pump use requires [57, 58]. Regional data indicate that device malfunction, infusion-set issues, and consequent suboptimal adherence can attenuate the glycemic benefit otherwise expected from CSII [59]. Potential solutions include structured reimbursement and national procurement programs to reduce cost barriers, investment in specialist multidisciplinary teams and structured patient/caregiver education, tele-diabetes support to extend specialist reach in under-resourced settings, and locally generated pragmatic effectiveness and cost-effectiveness studies, particularly of AID systems, to inform regional policy and improve QoL for children with type 1 diabetes and their families [57].

### Strengths

The primary strength of this study is its exclusive focus on RCTs and the application of TSA, which controls for type I and type II errors, a methodological advancement over prior reviews that often mixed observational data or failed to account for repetitive testing [7, 9].

### Limitations

High statistical heterogeneity (I^2^ = 84.5%) for HbA1c suggests that clinical diversity (e.g., age groups, insulin types, and pump models) and methodological differences (parallel vs. crossover designs) significantly influence outcomes. The open-label nature of insulin delivery trials introduces an unavoidable risk of performance bias, as reflected in our risk of bias assessment.

In addition, the rapid evolution of diabetes technology over the past decade, including ultra-rapid and second-generation basal insulin analogues, widespread continuous glucose monitoring, and hybrid and advanced hybrid closed-loop (AID) systems, has outpaced the evidence derived from the older pump and MDI regimens evaluated in most of the included trials [21, 55]. Contemporary pediatric RCTs of AID demonstrate clinically meaningful gains in time-in-range and in the proportion of children achieving HbA1c < 7% without increased hypoglycemia [60–62], indicating that the modest CSII-versus-MDI differences reported underestimate the benefit achievable with modern automated systems.

A further limitation is the age of much of the underlying evidence: eight of the 14 included RCTs were published more than 20 years ago, and many MDI control arms used NPH-based regimens that are no longer standard of care [4, 8]. The dearth of contemporary head-to-head pediatric RCTs is an important finding of this review, as the comparator of clinical interest has since shifted from conventional CSII toward AID [55, 60, 61]; therefore, the present synthesis should be interpreted as characterizing the legacy CSII-versus-MDI evidence base rather than current best-available technology.

While the field moves toward automation, understanding the continuing role of conventional pumps is essential. Exact population-wide prevalence data comparing total CSII versus AID use remain sparse; however, within pediatric technology cohorts, the proportion of AID use is rising rapidly, with one recent registry noting that nearly 73% of pediatric automated system users are utilizing newer, non-calibration AID generations [63].

Despite strong evidence that AIDs provide superior glycemic outcomes compared to non-automated pumps, conventional CSII retains important practical and situational advantages. Standard CSII is often preferred in clinical contexts where automation algorithms are unavailable, unwanted, or considered off-label [64]. Additionally, CSII carries lower upfront costs than advanced AID systems paired with continuous glucose monitors [65]. For many families, standard CSII offers a highly accessible, simplified technological step that improves treatment satisfaction without the added device burdens and algorithmic complexities associated with AIDs [66].

Also, the geographic distribution of the evidence is skewed as the included trials originate almost exclusively from the USA, Western/Northern Europe, and Israel, with no eligible pediatric RCT from the MENA region, Sub-Saharan Africa, Latin America, or East Asia [57, 58]. This limits the external validity of the findings for health systems with different reimbursement structures, device availability, and access to specialist support and underscores the need for trials and real-world evaluations in underrepresented populations.

### Conclusions

Current randomized evidence suggests that while CSII may offer a modest glycemic benefit over MDI in pediatric T1DM, this advantage is not definitive across all settings and does not come at the cost of increased safety risks. The choice between CSII and MDI should be shared, accounting for clinical goals, resource availability, and individual family preferences. Future research should prioritize large-scale RCTs comparing modern AID systems with state-of-the-art MDI regimens to maintain relevance in this rapidly advancing field. Notably, the 2026 ADA Standards of Care now recommend AID as the preferred insulin-delivery method for all people with type 1 diabetes; our TSA demonstrates why the conventional CSII-versus-MDI question never reached statistical resolution and reinforces that future research should compare modern AID systems against optimized MDI rather than re-examining legacy pump regimens. Future research should prioritize region-representative trials and cost-effectiveness analyses, especially in under-served populations such as the MENA region, to address barriers of cost, infrastructure, and ongoing support that currently limit equitable access to pump and AID therapy.

## Supplementary Material

Suppl 1 PRISMA 2020 checklist.

## Data Availability

All data analyzed are included in this article and its supplementary files. Search strategies and extraction forms are available from the corresponding author upon request.

## Abbreviations

BMI: body mass index
CGMS: continuous glucose monitoring system
CI: confidence interval
CSII: continuous subcutaneous insulin infusion
DCCT: Diabetes Control and Complications Trial
DKA: diabetic ketoacidosis
GRADE: Grades of Recommendations Assessment, Development, and Evaluation
HbA1c: glycated hemoglobin
HKSJ: Hartung–Knapp–Sidik–Jonkman
HRQoL: health-related quality of life
MD: mean difference
MDI: multiple daily injections
NPH: neutral protamine Hagedorn
PI: prediction interval
PRISMA: Preferred Reporting Items for Systematic Reviews and Meta-Analyses
QoL: quality of life
RCT: randomized controlled trial
REML: restricted maximum likelihood
RIS: required information size
RoB 2: risk of bias 2.0
RR: risk ratio
SMD: standardized mean difference
T1DM: type 1 diabetes mellitus
TDD: total daily dose
TSA: trial sequential analysis
